# Assessing experienced tranquillity through natural language processing and landscape ecology measures

**DOI:** 10.1007/s10980-020-01181-8

**Published:** 2021-01-27

**Authors:** Flurina M. Wartmann, Olga Koblet, Ross S. Purves

**Affiliations:** 1 School of Geosciences, University of Aberdeen, St Mary’s, Elphinstone Road, Aberdeen, AB24 3UF UK; 2 Swiss Federal Institute for Forest, Snow and Landscape Research (WSL), Zürcherstrasse 111, 8903 Birmensdorf, Switzerland; 3 Department of Geography, University of Zurich, Winterthurerstrasse 190, 8057 Zurich, Switzerland

**Keywords:** Tranquillity mapping, Landscape perception, Landscape quality, User-generated content, Natural language processing

## Abstract

**Context:**

Identifying tranquil areas is important for landscape planning and policy-making. Research demonstrated discrepancies between modelled potential tranquil areas and where people experience tranquillity based on field surveys. Because surveys are resource-intensive, user-generated text data offers potential for extracting where people experience tranquillity.

**Objectives:**

We explore and model the relationship between landscape ecological measures and experienced tranquillity extracted from user-generated text descriptions.

**Methods:**

Georeferenced, user-generated landscape descriptions from Geograph.UK were filtered using keywords related to tranquillity. We stratify resulting tranquil locations according to dominant land cover and quantify the influence of landscape characteristics including diversity and naturalness on explaining the presence of tranquillity. Finally, we apply natural language processing to identify terms linked to tranquillity keywords and compare the similarity of these terms across land cover classes.

**Results:**

Evaluation of potential keywords yielded six keywords associated with experienced tranquillity, resulting in 15,350 extracted tranquillity descriptions. The two most common land cover classes associated with tranquillity were *arable and horticulture*, and *improved grassland*, followed by *urban* and *suburban*. In the logistic regression model across all land cover classes, freshwater, elevation and naturalness were positive predictors of tranquillity. Built-up area was a negative predictor. Descriptions of tranquillity were most similar between *improved grassland* and *arable and horticulture,* and most dissimilar between *arable and horticulture* and *urban.*

**Conclusions:**

This study highlights the potential of applying natural language processing to extract experienced tranquillity from text, and demonstrates links between landscape ecological measures and tranquillity as a perceived landscape quality.

**Supplementary information:**

The online version of this article (10.1007/s10980-020-01181-8) contains supplementary material, which is available to authorized users.

## Introduction

Tranquil places, offering an escape from the pressures of everyday life, are increasingly recognised as a landscape quality that should be protected and accounted for in policy. Concepts related to tranquillity are included in the European Environmental Noise Directive (Nugent et al. 2016) and explicitly in national planning frameworks, for example in England (Ministry of Housing Communities and Local Government 2018):

‘For an area to justify being protected for its tranquillity, it is likely to be relatively undisturbed by noise from human sources that undermine the intrinsic character of the area. It may, for example, provide a sense of peace and quiet or a positive soundscape where natural sounds such as birdsong or flowing water are more prominent than background noise, e.g. from transport’ […] (§008, Reference ID: 30-008-20190722, updated 22nd of July 2019, https://www.gov.uk/guidance/noise--2).

Policies aiming to protect such locations highlight the need to identify, map and model tranquil areas, resulting from the interaction between landscape properties and human perception (Watts and Pheasant 2015). Identifying such areas requires integrating methods from the natural and social sciences. Models typically first explore factors adding to or diminishing tranquillity, through for example interviews, focus groups and literature studies. These factors are then operationalised in a Geographic Information System (GIS) to create continuous models of potential tranquillity (MacFarlane et al. 2004; Jackson et al. 2008; Hewlett et al. 2017). However, empirical work has found large differences between where individuals reported experiencing tranquillity and model predictions (Wartmann and Mackaness 2020). This suggests that tranquillity is not necessarily consumed where there is most supply (i.e. in the most tranquil areas), but rather where it is accessible to a broader population. We therefore refer to *potential tranquillity* where the potential for tranquillity exists, contrasting with *experienced tranquillity* where people go to appreciate it. Furthermore, descriptions of perceived sounds in different landscapes expose a dichotomy between small islands of experienced tranquillity contrasting with their surroundings, such as in urban green spaces, and regions of experienced tranquillity embedded within larger and more homogeneous settings of potential tranquillity (Chesnokova and Purves 2018). While tranquil regions in homogenous landscape settings map well onto results of current GIS-based modelling approaches for potential tranquillity, small pockets of experienced tranquillity described through contrast do not. Yet such spatially limited pockets of experienced tranquillity may be important for the provision of tranquillity as a landscape quality to populations in urbanised landscapes. This observation highlights the need to take into account landscape pattern and scale in combination with experienced tranquillity.

However, collecting in situ data on experienced tranquillity through surveys or questionnaires is resource-intensive, even for small study areas (South Downs National Park Authority 2017). Given this challenge, recent research has made use of user-generated content where people describe their experiences in landscape and share photographs and associated texts online. Using such sources allows studying human experience in landscapes across potentially large temporal and spatial scales (Chesnokova et al. 2019; Seresinhe et al. 2019). Although previous work has explored the importance of landscape features such as water bodies and green spaces for tranquillity (Herzog and Bosley 1992; Watts et al. 2013), the relationship between experienced tranquillity and landscape characteristics so far remains under investigated.

We explore here the relationship between different landscape measures and textual descriptions of experienced tranquillity, addressing the following research questions:

- RQ1: Do people experience and describe tranquillity differently in different environmental settings?
- RQ2: What factors influence experienced tranquillity in these different environmental settings?

To empirically address these research questions, we chose Great Britain as a study area. We did so for two reasons. Firstly, tranquillity has been explicitly recognised as a landscape property in policy nationally and locally in the UK (Ministry of Housing Communities and Local Government 2018; South Downs National Park Authority 2017). Secondly, there is also an extensive history of modelling tranquillity in the UK (MacFarlane et al. 2004; Jackson et al. 2008; Hewlett et al. 2017; Chesnokova and Purves 2018; Wartmann et al. 2019).

We investigate user-generated content in the form of texts linked to specific tranquil landscapes from the *Geograph* project, a crowdsourcing project aiming to collect georeferenced photographs and associated descriptions for every square kilometre in the British Isles. We use natural language processing (NLP) to analyse Geograph texts and link these to landscape characteristics. This hybrid methodology allows us to explore the links between where and how people experience tranquillity in relation to landscape characteristics related to properties including land cover, topography and human presence in landscapes (MacFarlane et al. 2004; Jackson et al. 2008). The novelty of our approach lies in combining natural language processing of textual descriptions and GIS data to explore people’s experience of tranquillity in different landscape settings*.*

### Background

Experiencing tranquillity offers relief from stress and contributes to well-being (Ulrich et al. 1991). Attention restoration theory postulates that recovery from stress, or cognitive overload, is best achieved in natural environments (Kaplan and Kaplan 1989). Based on this theory, Herzog and Bosley (1992) and Herzog and Barnes (1999) carried out empirical studies in which subjects had to rate images of different environmental settings according to their perceived tranquillity and personal preferences*.* Tranquillity was rated higher than preference for the categories ‘fields and forests’, ‘mountains’ as well as ‘rivers, lakes and ponds’. Subsequent experimental studies demonstrated that auditory and visual stimuli interact to create tranquil experiences, and highlighted the positive contribution of natural features and greenery (Watts et al. 2011; Watts 2017).

#### Modelling and mapping tranquillity

Existing models to predict tranquil areas are based on criteria such as distance from infrastructure and naturalness of land cover, which are mapped in a GIS, with criteria weighting based on public consultations (Hewlett et al. 2017; Jackson et al. 2008; MacFarlane et al. 2004). Resulting maps show potential for tranquillity, which may not be where most people go to ‘consume’ tranquillity. This challenge is similar to other approaches attempting to model and map human experience of landscape based on bio-physical landscape data, such as wilderness mapping (Fritz et al. 2000; Carver et al. 2009). Both for wilderness and tranquillity mapping, the results are not typically validated by testing whether individuals actually consider the mapped locations to be wild or tranquil. Where field-based public ratings have been compared to model results, contradictions are revealed. For instance, in a Scottish national park, respondents reported high levels of tranquillity despite the area being modelled as not tranquil due to presence of noise from busy roads (Wartmann and Mackaness 2020). Because scaling up field-based assessments of tranquillity is usually cost-prohibitive, there is a need for scalable methods to extract experienced tranquillity.

#### Extracting landscape experience from user-generated data

User-generated data such as geolocated images and associated descriptions contain information about how people experience landscapes (Gliozzo et al. 2016; Seresinhe et al. 2019). While there is prolific research on the use of georeferenced images (Seresinhe et al. 2017; Tenkanen et al. 2017), text data have so far been used less. We can distinguish two basic forms of text data: (i) text in the form of keywords or *tags* often associated with images, and (ii) full-text descriptions of varying length such as descriptions of images or travel blogs. Content in the form of tags is created when users of photo sharing platforms such as Flickr or Instagram apply tags to describe uploaded images so that other users may find and ‘like’ them. Users may also add coordinates to their images, thus linking the tags directly to a geographic location. This georeferencing enabled studies that extract user-generated images for a certain spatial extent and then characterise this area using the image tags (e.g. Capineri 2016). Another approach is to search for certain tags and then map the locations of images with these tags (e.g. van Zanten et al. 2016).

Research on tranquillity has also made use of image tags. By extracting images with tags semantically related to tranquillity and then mapping their locations experienced tranquillity in a Scottish national park could be mapped (Wartmann and Mackaness 2020). Scaling-up this approach to all of Scotland and integrating automated-image processing to filter landscape images demonstrated that this approach could indicate hotspots of experienced tranquillity across a large spatial extent (Wartmann et al. 2019).

The advantage of using tags as a text-source is that they can be easily extracted and processed. However, full-text descriptions contain richer semantics and can analysed manually through detailed qualitative and quantitative analysis (Bieling 2014). Such approaches are though resource-intensive, limiting the sample size and spatial extent of the study. To overcome this limitation, computational methods such as natural language processing (Manning and Schütze 1999) can be used to analyse large volumes of text. For example, a corpus of historic writings on the English Lake District was analysed to extract and characterise aesthetic landscape experiences (Donaldson et al. 2017). Using the same corpus, Chesnokova et al. (2019) compared historical to modern descriptions of soundscapes. Although research exploring tranquillity through text-based approaches shows potential, these data have not been combined with landscape ecological measures to assess the influence of landscape composition on tranquillity.

## Methods

**Fig. 1:**
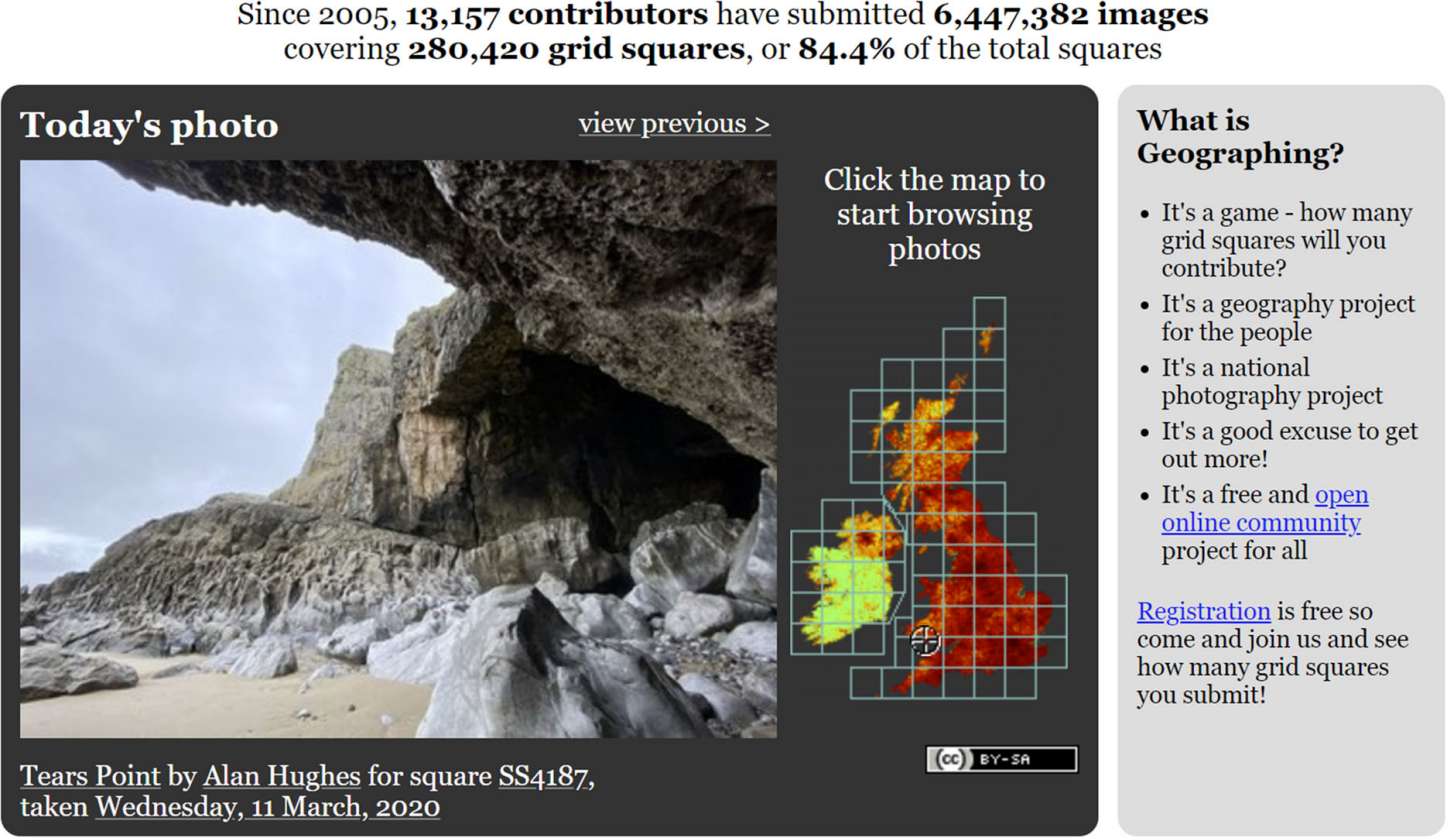
Interface of Geograph. Red colours on the map demonstrate the density of the contributions, green colour shows ‘empty’ squares

**Fig. 2:**
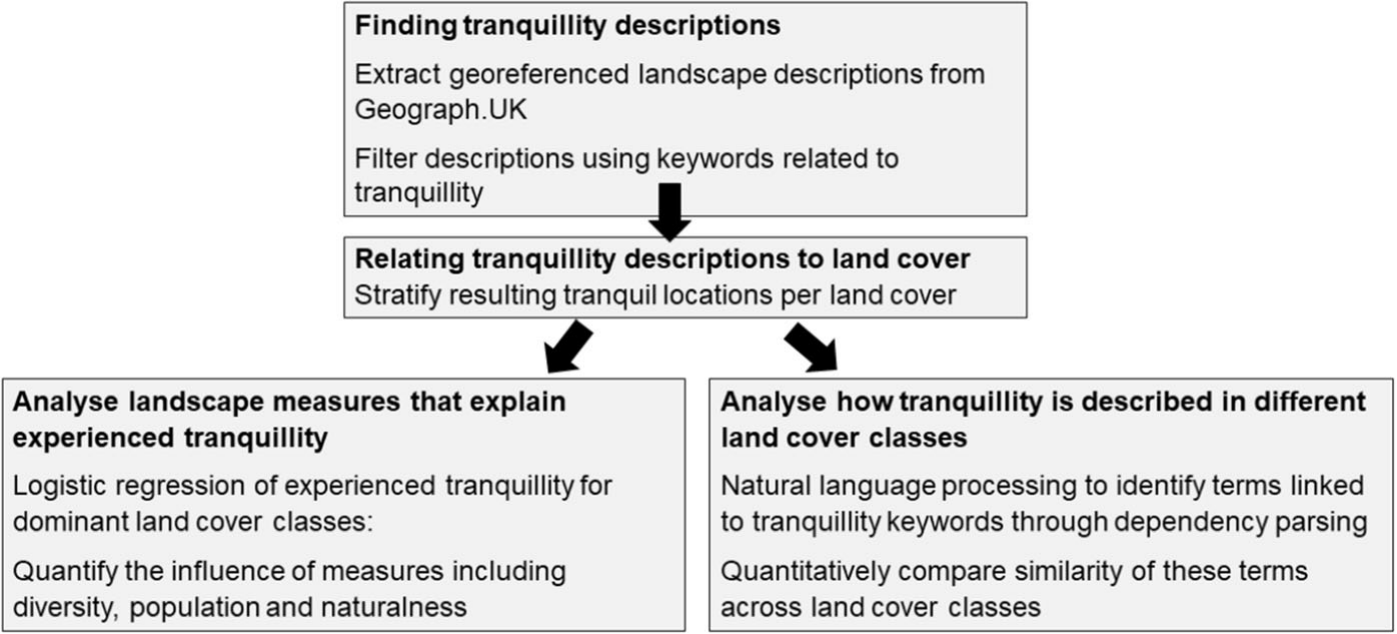
Overview of methodological approach

We harvested text in the form of georeferenced landscape descriptions from the Geograph platform (Fig. 1). These descriptions accompany landscape photographs, where the declared goal is to photograph and describe every square kilometre grid cell in the British Isles. Using a combination of qualitative and quantitative methods, we extracted and analysed descriptions of tranquillity from Geograph.UK (Fig. 2). We focused on experienced tranquillity by extracting only descriptions containing keywords semantically related to tranquillity, refining our selection through a combination of manual and automated annotation. We then stratified our text data according to the dominant land cover class for the location referred to in the description. To assess the influence of different landscape ecological measures, such as diversity or naturalness, in explaining the presence or absence of tranquillity, we used a logistic regression model. We then explored reasons for differences in model results by comparing quantitatively and qualitatively similarity between descriptions in different land cover classes.

### Study area

**Fig. 3:**
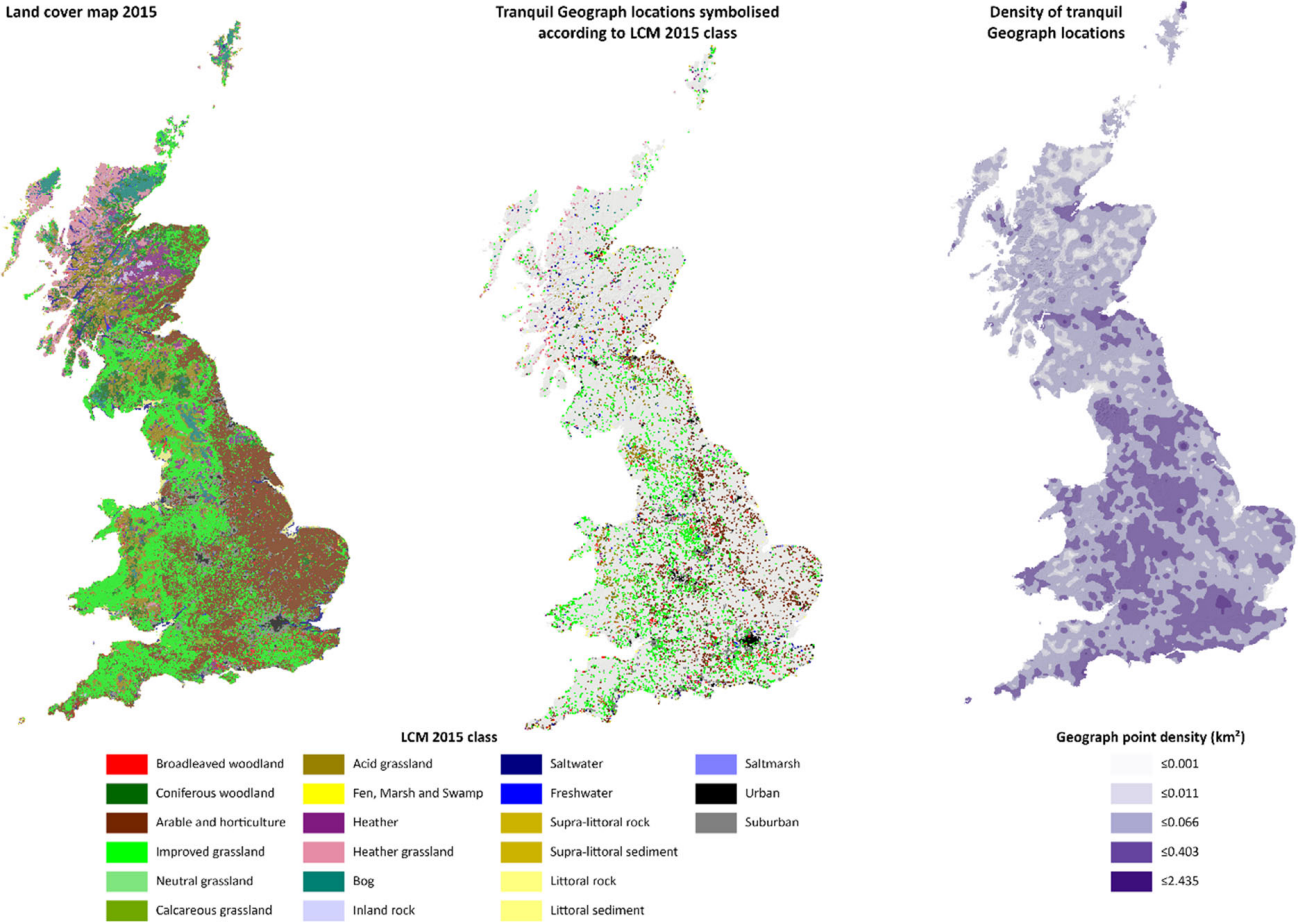
Distribution of LCM15 land cover classes, individual Geograph descriptions containing tranquillity related descriptions coloured according to LCM15 class and density per km^2^ of Geograph descriptions containing tranquillity

The landscapes and habitats of Great Britain are varied, ranging from coastal habitats to remote mountain landscapes (Lake et al. 2015) (Fig. 3). Broad habitat types include woodland, grassland, wetland and freshwater, classified using a range of different systems. For instance, the ‘Broad Habitat Types’ that serve as a basis for UK habitat mapping and land cover data classification, such as in the ‘Land Cover Map’ (CEH 2017), which we used for this study.

### Data sources

We used two main data sources, text data and biophysical data on landscapes in the Great Britain, which we describe in more detail in the following.

#### The Geograph project

Geograph^1^ was launched in 2005 and has been used in a range of landscape-related research, including studies on the influence of scenic landscapes on health (Seresinhe et al. 2015) and creation of landscape sentiment lexicons (Koblet and Purves 2020). Its unique feature is a collection of representative photographs and textual descriptions of the British Isles, gathered through a game-like approach, where authors can turn a ‘green’ (empty) square into a ‘red’ (occupied) one (Fig. 1). The collection currently contains almost 6.5 million photographs contributed by more than 13,000 authors. The dataset is available for download under a Creative Commons License.

#### Land cover, population and topography

In order to relate experienced tranquillity to land cover across our study area we use the following raw data:

- Land Cover Map 2015 (LCM15), published in 2017 by the Centre for Ecology and Hydrology (CEH 2017). The LCM15 classifies primarily Landsat 30 m data into 21 classes, based on the UK Biodiversity Action Plan Broad Habitat definitions (Jackson 2000). LCM15 is available as a vector product and a 25 m raster, from which the two input products we used were derived. These have a resolution of 1 km, representing respectively the dominant land cover class (Rowland et al. 2017a), and the percentage of a 1 km grid cell taken up by each of the 21 classes (Rowland et al. 2017b).
  To capture other potentially important spatial variables related to tranquillity we used three further datasets:
- Global Human Settlement Population (GHS-POP), which estimates the number of residents in a 250 m grid cell in 2015 (Corbane et al. 2018)
- Global Human Settlement (GHS-BUILT) which estimates the percentage of a 250 m grid cell defined as built-up based on Landsat data from 2013/14 (Schiavina et al. 2019).
- 250 m resolution digital elevation model (DEM) from the ESRI World Elevation Terrain Service (ESRI 2012).

These raw data were then used to derive the independent variables described in 2.3.2 used as predictors of tranquillity.

### Analysis

#### Analysing experienced tranquillity in landscape descriptions from Geograph data

We started with the 12 search terms related to tranquillity used by Wartmann et al. (2019) *atmosphere, calmness, peace, peaceful, pleasant, serene, tranquillity, tranquil, silence, silent, quiet*. These terms were derived from interviews with visitors to a national park in Scotland describing what contributed to their experience of tranquillity (Wartmann and Mackaness 2020). To this set of terms, we added an alternative spelling for tranquillity: *tranquillity* and added an additional synonyms for calmness: *calm.* The full set of terms used in our analysis was thus: *tranquillity, tranquility, tranquil, silence, silent, peace, peaceful, serene, quiet, calmness, calm, pleasant, atmosphere*. To gauge the efficacy of our term list for selecting locations where users experienced tranquillity, we first annotated descriptions containing these terms as either ‘experienced tranquillity’ or ‘not related to tranquillity’. To do so we extracted 100 random descriptions using each of the search terms, resulting in 1300 landscape descriptions containing the search terms.^2^ However, not all descriptions containing our keywords are necessarily related to experienced tranquillity. Therefore, based on our initial set of random descriptions, we developed a set of rules that allowed us to classify descriptions as ‘tranquillity’ or ‘not relevant’. We evaluated the consistency of our annotation rules using Cohen’s Kappa (Landis and Koch 1977) as the inter-annotator agreement measure for a random selection of 100 out of the 1300 descriptions. We then used the results from the manual annotation of our descriptions to reduce our set of search terms to those that yielded high percentages of descriptions about experienced tranquillity. From the remaining descriptions, we extracted coordinates and created a point data set of locations where Geograph users describe experienced tranquillity.

#### Logistic regression of experienced tranquillity for land cover classes

Having used text-based data to identify locations where people describe experienced tranquillity, we now link and analyse these locations in relation to dominant land cover classes.

Since potential tranquillity is argued to be a function of a variety of landscape properties, including those related to land cover, topography and human presence in landscapes (MacFarlane et al. 2004; Jackson et al. 2008), we explored how much such variables were able to explain the experienced tranquillity locations captured through our text data. To do so we used a general linear model with tranquillity as a binary dependent variable. To represent non-tranquil locations, we randomly extracted around 15,000 descriptions from the Geograph dataset, which did not contain any of the final set of search terms. Attributes for all dependent variables were extracted from raster data values at the point locations. As independent variables we included the following:

- *Water* is often considered to be an essential element of tranquil landscapes and has been empirically shown to be related to what people perceive as tranquil (Herzog and Bosley 1992; Wartmann et al. 2019). LCM15 contains two water related classes, fresh and salt water. Salt water is especially relevant in Scotland, since many sea lochs (fjords) are tidal. We included the percentage of fresh and salt water in a 1 km cell.
- *Perceived naturalness*, in terms of the mix of land cover classes, has been argued to be an important element of tranquil landscapes. We calculated perceived naturalness following the approach proposed by MacFarlane et al. (2004), who, based on empirical work with public consultations and photo ratings, assigned weights to individual land cover classes, and calculated the weighted sum of the percentage land cover in each grid cell. Since the classes used in LCM15 differed slightly from those used by MacFarlane et al. (2004) we mapped classes to their most similar counterparts in the original data.
- *Diversity* in land cover is suggested to be a predictor of aesthetically pleasing landscapes, and in turn tranquillity. We calculated diversity using the Shannon–Weaver index (Ortiz-Burgos 2016), as:
  $$\mathrm{H}=-{\sum }_{i=1}^{S}{p}_{i} ln{p}_{i}$$

where *p*_*i*_ is the proportion of LCM15 land cover class *i* found in a 1 km grid cell and *S* is the number of land cover classes found in a 1 km grid cell.

- *Presence of people* is often reported as detracting from tranquillity. We used the absolute population in a 250 m grid cell (GHS-POP) as a proxy for the presence of people.
- *Built-up areas*, not only in the form of buildings but also roads and other built infrastructure have been found to detract from tranquillity (Jackson et al. 2008; MacFarlane et al. 2004). Furthermore, data on built-up areas may be an effective way of capturing pockets of tranquillity in parks and other urban green areas. We therefore included the percentage of built-up area within a 250 m grid cell (GHS-BUILT).
- *Upland areas* in Great Britain are generally associated with more remote locations, and thus tranquillity (Jackson et al. 2008; MacFarlane et al. 2004). We thus included absolute elevation at a 250 m resolution.
- *Terrain roughness* is related to wilderness (Carver et al. 2002), and empirical research has shown mountainous environments to be rated as highly tranquil (Herzog and Bosley 1992). We calculated terrain roughness according to the vector ruggedness model proposed by Sappington, Longshore and Thompson (Sappington et al. 2007) in a 7 × 7 window at a resolution of 250 m.
- Finally, we included *latitude* and *longitude* since we expected the nature of tranquillity to vary across west–east and north–south gradients.

We used the GLM package in R^3^ to explore the relation between experienced tranquillity and the independent variables described above. We first created a global model, containing all tranquil locations we found in Great Britain and the random dataset of non-tranquil locations described above. A second set of stratified models then explored the relationship of tranquillity to selected land cover classes (termed hereafter as forest, agriculture, improved grassland and urban models).

All models used identical independent variables and we calculated the variance inflation factor for all independent variables to check for autocorrelation. For each model we calculated Hosmer and Lemeshow goodness of fit to determine whether the modelled fit to deciles of tranquil/ non-tranquil locations was similar to that we observed in the original data (Hosmer et al. 1988).

#### Exploring landscape descriptions related to tranquillity in different land cover classes using natural language processing

After linking locations described as tranquil to spatial data using a GLM, we use natural language processing to look in more detail at what kind of objects or concepts these terms describe and how they vary according to land cover classes. Using the natural language processing python library spaCy,^4^ we identified all nouns modified by adjectives linked to tranquillity. For example, from the sentence ‘I enjoyed this tranquil and peaceful spot’ we extracted *tranquil spot* and *peaceful spot*. To assess which land covers are described more similarly to one another, we calculated cosine similarities (Manning and Schütze 1999) as a measure of similarity between the descriptions for different land cover classes. Cosine similarity was calculated by representing all nouns found in descriptions of tranquillity for one land cover class as a vector of N terms, where N is the number of terms appearing in the corpus and each term is weighted by its frequency of occurrence in the respective document. The similarity between descriptions was then calculated as the cosine between this and a second vector (Manning and Schütze 1999), e.g. between the vector for *urban* and the vector for *broadleaved woodland*.

## Results

### Defining appropriate search terms to extract descriptions of tranquillity

For each of our list of 13 search terms we aimed to extract 100 random descriptions from the Geograph collection. However, as the word *calmness* was used only 16 times in total, the total dataset consisted of only 1216 descriptions. Of those, we selected 100 random descriptions to test our annotation rules with two authors, resulting in a Cohen’s Kappa of 0.880, p < 0.001, considered an (almost) perfect agreement (Landis and Koch 1977).

Table 1 shows differences in how many of the descriptions actually referred to experienced tranquillity. The term most consistently used in referring to tranquil landscape settings was *quiet* with 90% of examples in our sample, followed by *tranquil, peaceful* and *calm*. Search terms that proved too semantically ambiguous to be useful in identifying descriptions of tranquil landscape settings included ‘atmosphere’, with 94% of examples referring to other contexts, e.g. ‘lively atmosphere’, ‘welcoming atmosphere’ or ‘forlorn atmosphere’. The ambiguity also stems from the use of the term atmosphere to refer to the celestial atmosphere, e.g. ‘Residents in the UK were promised spectacular sunsets as a result of ash in the upper atmosphere.’^5^ The term ‘peace’ was often used to refer to peace in the context of war and conflict, and as part of place names, and not as a feeling associated with landscape. For instance, ‘Part of the City Hall, viewed from the Peace Gardens across the Goodwin Fountain.’^6^ Other examples include references to historic events: ‘The OS map captions the shop as the Royal Oak (PH), and I am informed that it is indeed a public house, although I couldn't see an inn sign. Over the door is the inscription “The last invasion of Britain peace treaty was signed here in 1797”.’^7^

**Table 1:** Results of the manual annotation of 1216 randomly selected descriptions containing our initial search terms

Keyword	Found to be used in description not related to tranquillity	Found in description related to tranquillity	Number of examples
atmosphere	94	6	100
calm	20	80	100
calmness	8	8	16
peace	92	8	100
peaceful	19	81	100
pleasant	63	37	100
quiet	10	90	100
serene	25	75	100
silence	68	32	100
silent	90	10	100
tranquil	16	84	100
tranquility	23	77	100
tranquillity	36	64	100
Total number	564	652	1216

Based on our content analysis, we retained four terms that were most often associated with tranquil descriptions, *tranquil, quiet, peaceful* and *calm* as well as the spelling variations of the nouns *tranquillity* and *tranquility* to extract full text descriptions and associated locations.

Using the final set of 6 search terms we extracted 15,904 descriptions. To reduce bias introduced by individual contributors (Hollenstein and Purves 2010) we retained one random description per author if the descriptions of several images were identical. This filtering step resulted in 15,350 descriptions contributed by 1397 unique authors.

### Mapping and characterising experienced tranquillity

**Fig. 4:**
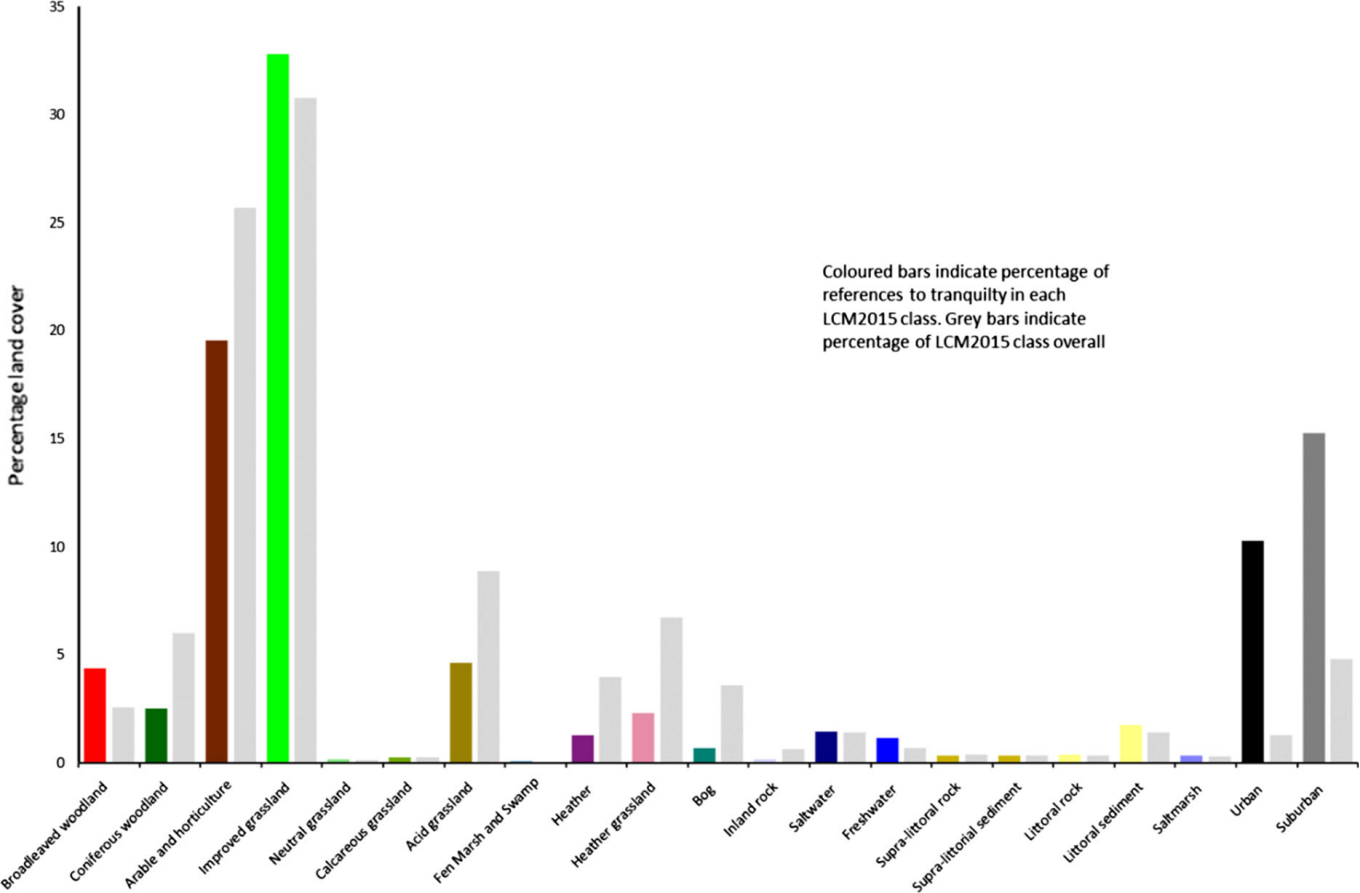
Differences of percentage of tranquillity description found per land cover class in relation to overall proportion of land cover classes

Figure 3 gives an overview of the distribution of LCM15 land cover classes in Great Britain (Fig. 3 left), the point locations associated with experienced tranquillity (Fig. 3 middle) and the density of these point descriptions (Fig. 3 right). Figure 4 shows the percentages of tranquillity descriptions associated with each land cover class, along with the overall percentage of these land cover classes. The two most common land cover classes associated with tranquillity are *arable and horticulture* and *improved grassland* respectively. However, the proportion of descriptions associated with *improved grassland* is more than expected based on the area represented by this landcover class (32.8% of tranquil locations are improved grassland vs. 30.8% of the total landcover in this class), while *arable and horticulture* is underrepresented (19.5% vs. 25.7%). This is also reflected on the map of tranquillity locations, with the widespread distribution of experienced tranquillity in the west. The next most common classes of experienced tranquillity are *urban* and *suburban*. Here, tranquil locations are many times more common than we would expect based on the area represented by these classes (*urban* 10.3% vs. 1.3% and *suburban* 15.3% vs. 4.8%). This likely reflects on the one hand, the overall distribution of user-generated descriptions, with clustering in urban areas (Antoniou et al. 2010), and on the other a need for tranquillity in these populated settings. The next most common class is *acid grassland*, though here only around half as many tranquil experiences are reported as might be expected based on area (4.6% vs. 8.9%). By contrast, broadleaved woodland has almost twice as many reports of experienced tranquillity as we might expect (4.4% vs. 2.6%) with coniferous woodland being much less likely to be associated with tranquillity (2.5% vs. 6%). These distributions are reflected in the density surface (Fig. 3 right) which shows that experienced tranquillity is much more associated with more populated locations, such as the Scottish Central belt and England in general, popular tourist attractions (e.g. the Lake District National Park in England) and urban centres (e.g. London).

In the following, we look in more detail at six land cover classes with the highest counts of photographs described with a keyword related to tranquillity: broadleaved woodland, coniferous woodland, arable and horticulture, improved grassland, urban and suburban (Table 2).

**Table 2:** Number of tranquillity descriptions and unique authors per land cover class

Class	Number of descriptions	Number of unique authors
Broadleaved woodland	399	196
Coniferous woodland	218	101
Arable and horticulture	1987	434
Improved grassland	3236	602
Urban	900	263
Suburban	1494	388

### Using landscape characteristics to model experienced tranquillity

To test different predictors, we calculated logistic regression models for a global model across all land covers and for four land-cover-based models. For the two most common classes (*arable and horticulture* and *improved grassland*) we created individual models. We grouped *broadleaved* and *coniferous woodland* and *urban* and *suburban* locations to allow larger sample sizes in what we hypothesised might be semantically similar tranquil experiences.

In the global model, significant positive predictors of tranquillity were increasing proportion of the land cover class freshwater and lower elevations (Table 3). Furthermore, higher values of naturalness were significant, whereas built-up area was a significant negative predictor. The Hosmer–Lemeshow goodness of fit test indicates that the global model adequately fits the data (p > 0.05).

**Table 3:** Statistical modelling results for the logistic regressions (Z-values)

	Global	Combined woodland	Arable & horticultural	Improved grassland	Combined urban
Intercept	− 1.501 p = 0.13	− 1.074 p = 0.28	− 2.588 p < 0.01**	− 2.537 p < 0.05*	1.026 p = 0.30
Saltwater	0.272 p = 0.79	0.864 p = 0.39	− 0.246 p = 0.81	0.450 p = 0.65	0.638 p = 0.52
Freshwater	6.757 p < 0.001***	3.102 p < 0.01**	3.556 p = 0***	4.302 p < 0.001***	− 0.533 p = 0.59
Naturalness	3.136 p < 0.01**	3.304 p < 0.001***	2.891 p < 0.01**	0.972 p = 0.33	− 0.057 p = 0.95
Diversity	1.574 p = 0.12	2.952 p < 0.01**	1.301 p = 0.19	0.617 p = 0.54	− 1.520 p = 0.13
Population	1.506 p = 0.13	0.042 p = 0.97	− 1.050 p = 0.29	0.631 p = 0.53	0.677 p = 0.50
Built-up area	− 4.153 p < 0.001***	− 0.011 p = 0.99	1.247 p = 0.21	− 2.290 p = 0.022*	− 4.752 p < 0.001***
Elevation	− 5.316 p < 0.001***	− 0.483 p = 0.63	0.615 p = 0.54	− 0.978 p = 0.33	− 1.978 p < 0.05*
Roughness	1.177 p = 0.24	− 0.703 p = 0.48	− 0.600 p = 0.55	0.386 p = 0.70	− 1.916 p = 0.06
Latitude	1.463 p = 0.14	0.243 p = 0.81	1.849 p = 0.06	2.765 p < 0.01**	− 0.513 p = 0.61
Longitude	− 1.407 p = 0.16	0.922 p = 0.36	− 2.468 p < 0.05*	0.342 p = 0.73	− 0.266 p = 0.79
Df	28,057	1918	5792	8984	7785
Hosmer and Lemeshow goodness of fit	χ^2^ = 11.248, df = 9, p = 0.259	χ^2^ = 18.548, df = 9, p = 0.0293	χ^2^ = 12.723, df = 9, p = 0.176	χ^2^ = 12.558, df = 9, p = 0.184	χ^2^ = 20.528, df = 9, p = 0.0149

Focusing on the logistic regression models for models stratified by land cover, we found that freshwater was a positive predictor in the *combined woodland*, *agriculture and horticulture*, and *improved grassland* models, but not for *combined urban*. For *combined woodland* diversity, naturalness and freshwater were all significant predictors of experienced tranquillity. However, the Hosmer–Lemeshow test indicates lack of model fit for the forest model. For *arable and horticulture* landscape class, model fit was assessed as satisfactory and naturalness was again a positive predictor in this model, but not diversity. Geographical longitude is a significant negative predictor in the *arable and horticulture* model, indicating that western locations are described more as tranquil than eastern agricultural areas. The *improved grassland* model fits the data well, with freshwater and latitude significant positive predictors, and built-up area as a negative predictor. This result indicates a north–south gradient in how grasslands are described, and that built-up areas negatively affect where we find tranquillity descriptions in grasslands. The combined urban model showed a lack of model fit, and only ‘elevation’ was a significant (negative) predictor of tranquillity in this model.

### Semantics of tranquillity

Our exploration of experienced tranquillity using landscape characteristics demonstrated that for all classes we find significant predictors related to theory, and that both a global model and those for *arable and horticultural* and *improved grassland* fitted the data well. For the two combined models, though significant predictors were identified, the overall model fit was poor. Since experienced tranquillity was captured using textual descriptions, we now return to these through nouns identified by the dependency parser as being related to tranquil keywords and explore their relationship to landscape characteristics and theory.

Table 4 shows how similar individual land cover classes are in terms of the words used to describe each class using cosine similarity. The highest values of similarity are found for *arable and horticulture* and *improved grassland*, indicating that these two types of experienced tranquillity are described using similar terms. *Urban* locations are most different to these land cover classes, and most similar to *suburban* locations. *Broadleaved woodland* is semantically more similar to *arable and horticulture* and *improved grassland* than *coniferous woodland*, indicating that the nature of experienced tranquillity described by these two classes is quite different.

**Table 4:**
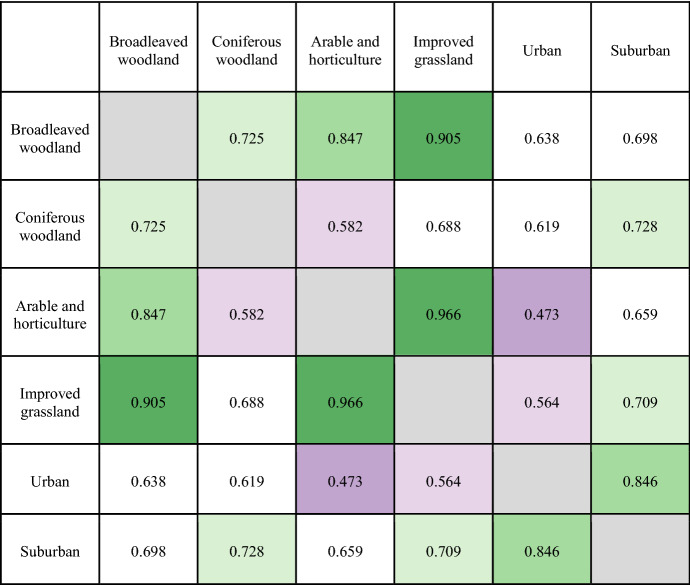
Cosine similarity between different land cover classes using nouns modified by tranquillity-related adjectives (e.g., ‘tranquil spot’) as an input. The matrix is symmetric. Dark green indicates high text similarity, dark purple low text similarity

**Fig. 5:**
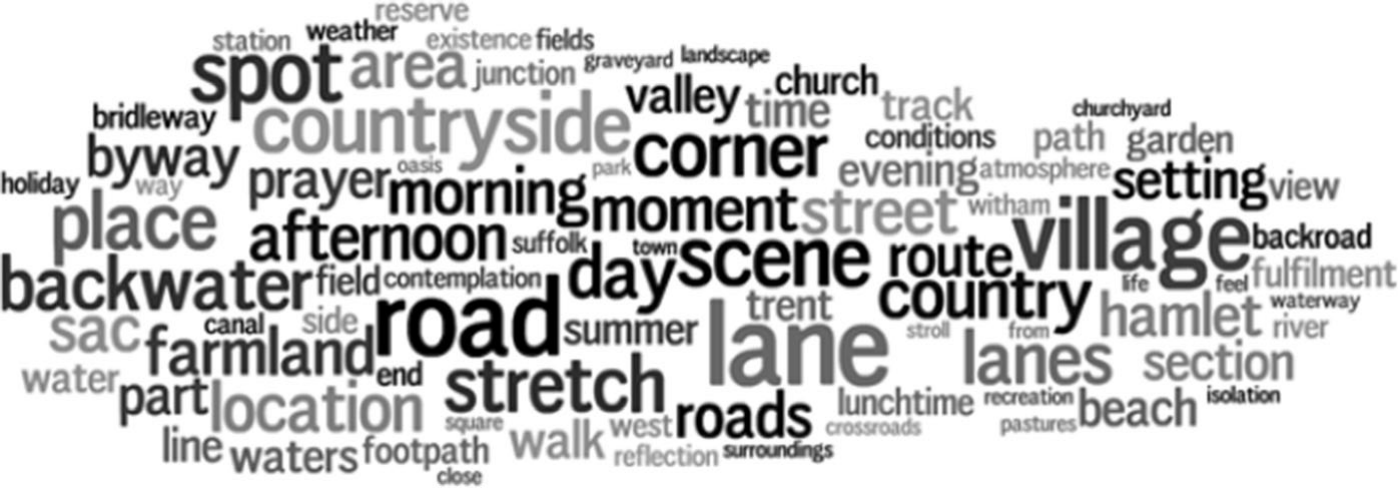
Word cloud for the land cover class ‘arable and horticulture’ showing terms identified through dependency parser to relate to tranquil keywords (font size relates to frequency, colours are for illustrative purpose only, number of unique words with frequency greater than 3 = 88)

This overview suggests that experienced tranquillity is described in different ways, but to understand how, we need to zoom in further. To do so, we created word clouds for each land cover class that show the frequency of nouns modified by our search terms, for example ‘quiet spot’ or ‘tranquil place’. Two of these (*arable and horticulture* and *urban)* are shown as exemplars in Fig. 5 (word clouds for all classes are available in the supplementary materials). Immediately apparent are the predominance of references to locations through generic terms (e.g., *place, spot, scene, area, setting*), to particular times (e.g., *morning, evening, day*) and paths through the landscape (e.g., *street, road, path*). In the *arable and horticultural* word cloud we also see references to its rural setting (e.g., *farmland, countryside, village* or *beach*) contrasting with what appear to be patches of tranquility in the urban word cloud (e.g., *haven, enclave, oasis*). By zooming in further, to actual descriptions, we can confirm that authors are indeed writing about an experience contrasted to its surroundings, and valued for this:

- “A quiet oasis away from the busy streets.”^8^
- “Many of the old lanes are preserved within the medieval street pattern of Totnes town centre. Atherton Lane is an oasis of calm just yards from the main street.”^9^
- “A welcome peaceful enclave.”^10^

These descriptions also suggest why the model for the *combined urban* land cover classes performed poorly. The finest resolution data we used had resolutions of 250 m, too coarse to capture locations described and illustrated by the images shown in the links to the photographed sites.

**Fig. 6:**
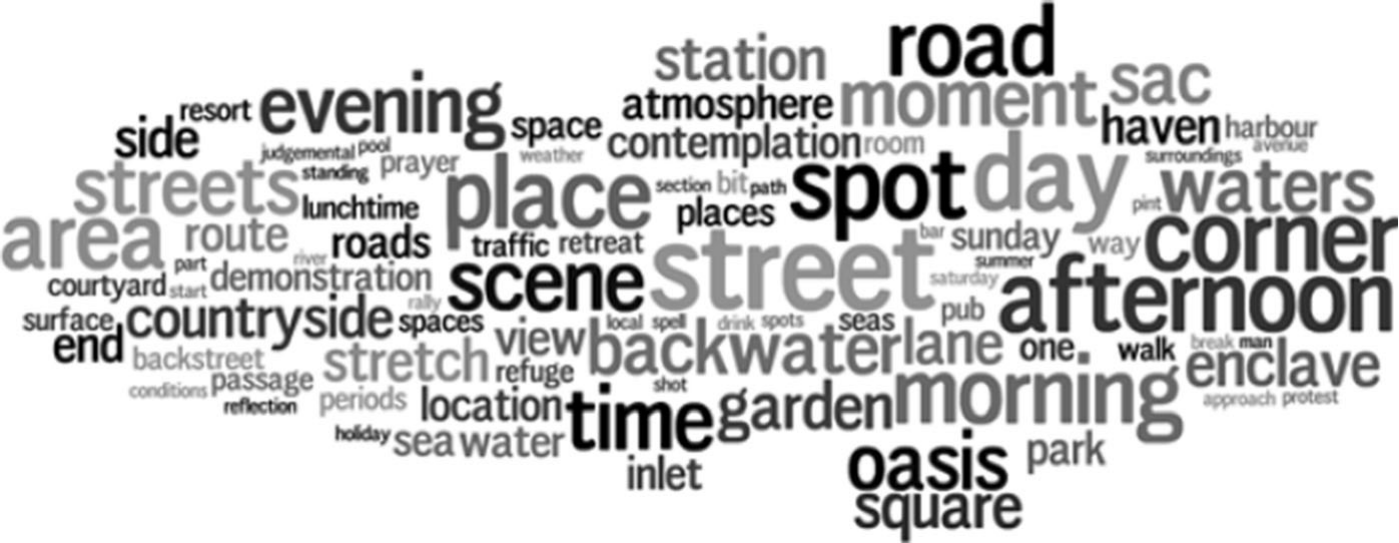
Word cloud for the land cover class ‘urban’ showing terms identified through dependency parser to relate to tranquil keywords (font size relates to frequency, colours are for illustrative purpose only, number of unique words with frequency greater than 3 = 63)

**Fig. 7:**
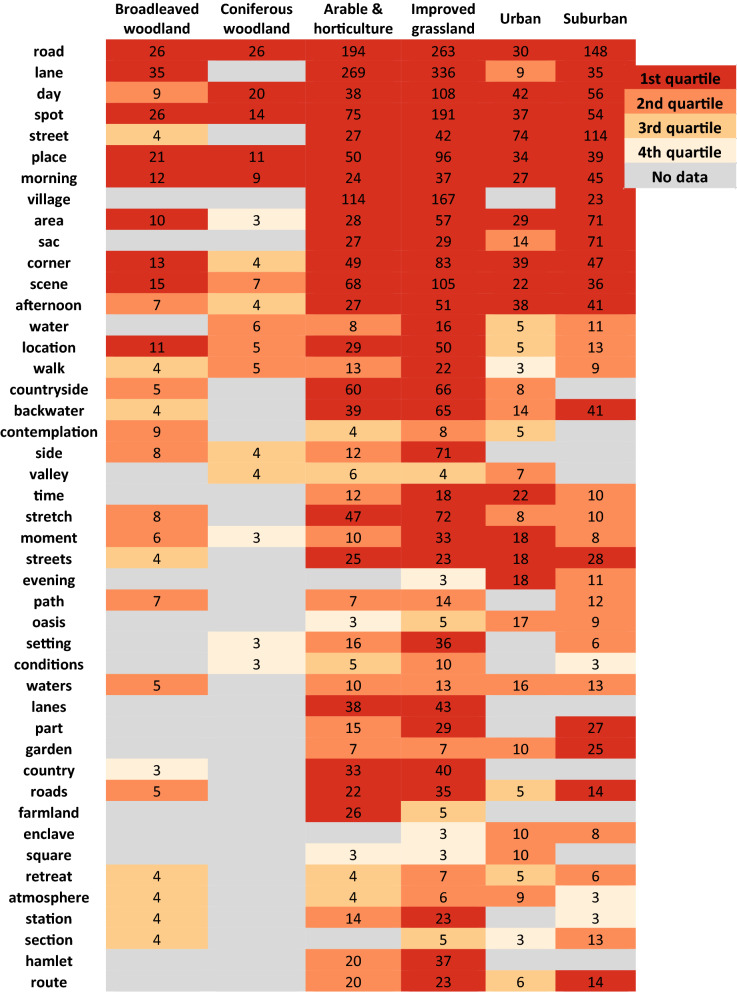
Most prominent terms used to describe the 6 land cover classes. Counts are total frequency of noun found, colours indicate to which quartile in each land cover class a term belongs. All terms with a frequency of more than ~ 1% in at least one land cover class are shown

Word clouds such as those in Figs. 5 and 6 are an excellent way of getting a sense of the semantics used to describe experienced tranquillity for a given land cover class. However, they are difficult to compare, and Fig. 7 attempts to fill this gap. Here, we show the most popular terms for each class, along with absolute counts and colour coded according to their quartile within a class. Thus, for example, we see that tranquillity related terms are associated with *road* in all six settings, contrasting directly with most models of potential tranquillity. The presence of a *village* is clearly an important indicator of experienced tranquillity in rural, non-wooded settings, and suburban locations. *Water*, despite its importance in theory, and its prominence in our models, is not a particularly popular way of describing such locations. Once again, we note the importance of time (e.g., *day*, *morning*) across all classes of land cover, and the idea of generically tranquil settings (e.g. *spot*, *place*, *area*, *corner*, *scene*).

## Discussion

In this study, we link landscape characteristics to how *people* perceive landscape quality, taking the example of tranquillity as the subjectively perceived ability of an environment to induce mental and physical relaxation. Building on a dataset of user-generated descriptions from the Geograph project allowed us to make use of unstructured text specifically created by a large number of people to describe landscapes in the UK.

### Using text descriptions to explore tranquillity as a landscape quality

Previous research analysed tag-based user-generated content, where photographs tagged with single keywords related to landscape were mapped (van Zanten et al. 2016; Wartmann et al. 2019), but ignored potential ambiguity. To reduce the effects of ambiguity in selecting descriptions we annotated over a thousand statements containing possible tranquillity keywords and retained only keywords which were often relevant. Analysing the semantically richer full-text data allows us better to explore words in context and thus deal with ambiguity.

Tranquillity descriptions were more associated with populated urban and suburban areas, where the frequency of descriptions was much higher than would be expected. In certain land cover types such as heather and bog, we found less tranquil locations than we would expect based on the percentage coverage of these land cover types. Mapping locations described as tranquil also highlights clusters of descriptions in iconic places and landscapes, which links well to previous results (Chesnokova and Purves 2018; Tenerelli et al. 2016). But these areas are fundamentally different from where models of potential tranquillity indicate that the most tranquil landscapes can be found (CPRE 2007; EEA 2016; MacFarlane et al. 2004). This observation accords with previous findings comparing quiet areas and field surveys on tranquillity, namely tranquillity is often experienced not in the most undisturbed locations, but rather in accessible areas with some potential for tranquillity, such as car parks and laybys offering scenic views (Wartmann and Mackaness 2020). Modelling hypothesised predictor variables in logistic regression models stratified for different land cover classes we saw that predictors differ between models for different land cover classes. For example, freshwater was a predictor of tranquil descriptions in woodlands, agricultural and grassland, but not in urban/suburban land cover. Using natural language processing allowed us to examine these results more closely. The grammatical dependencies of the terms related to our tranquillity descriptors showed that in urban environments, different elements are associated with experienced tranquillity to those in more rural or natural environments. This finding is important, because it indicates that different environments and different landscape elements can lead to a different, but equally valid, experience of tranquillity, challenging the notion that one model is suitable to predict tranquillity across large areas (Jackson et al. 2008; Hewlett et al. 2017). It thus illustrates the need for integrated approaches to user-generated content analysis combining data sources and analytical approaches (Heikinheimo et al. 2020; Jeawak et al. 2020).

The analysis of text offers opportunities for research on people-landscape interactions across potentially large spatial and temporal scales that are often difficult to explore using more traditional social science engagement methods such as surveys or field-based interviews. However, there are also limitations associated with such data. First and foremost, the descriptions were not written by a representative sample of the population, but a group (e.g. people willing and dedicated to take landscape photographs and descriptions to upload to Geograph), resulting in an unknown bias with respect to the part of the population represented. Although surveys often suffer from a response-bias, demographic data can determine that bias, which is more difficult for user-generated data. Equally, questions have been raised about the degree to which user-generated content is reflective of people’s more general offline behaviour (Sloan and Morgan 2015). In the case of Geograph, we argue that these data are digital traces of users who visited and described these locations, allowing us to link spatial data with a content analysis of these texts encapsulating people’s experiences in landscapes.

### Relating pattern, process and scale to tranquillity

Pattern, process and scale are fundamental concepts in landscape ecology (Turner 2005), and in the following, we relate our findings to these concepts. The research questions posed initially were: Do people experience and describe tranquillity differently in different environmental settings (RQ1)? And what factors influence experienced tranquillity in these different environmental settings (RQ2)? This study showed that experienced tranquillity as described in user-generated content is related to landscape ecological measures of landscape composition and differs across land cover classes. This finding suggests that the relation between land cover and experienced tranquillity is different to that typically modelled when mapping potential tranquillity in GIS. Models applying multi-criteria analysis of spatial data typically make no distinction between different settings such as urban or rural landscapes. For example, the same distance criteria are applied to features such as roads in different land covers or landscape settings (MacFarlane et al. 2004; Jackson et al. 2008; Hewlett et al. 2017). Zooming into different land cover classes as a proxy for different landscape settings highlights that in different types of land cover, different factors impact experienced tranquillity. We argue that this relates to the importance assigned to pattern in landscape ecology, and show that according to the setting, the composition of the landscape impacts differently on how people perceive landscape. This in turn also suggests that the process of how people perceive tranquillity is different for different settings, or places. We find evidence in the differences between terms used to describe tranquillity in different land cover classes. Our results showed that the terms grammatically related to (i.e. dependent on) our tranquillity keywords differ markedly between urban and suburban classes and agricultural / forested land cover classes. Finally, scale is also important. By exploring the semantics associated with tranquillity descriptions from many different individuals, we see that, for example, urban tranquillity is experienced at much finer spatial scales than tranquillity in agricultural or forest land cover. This difference in scales indicates we may need to model tranquillity differently according to the pattern and process involved in creating a tranquil experience, thus making it impossible to model at the same resolution across space. For example, modelling urban tranquillity, we need to consider much finer spatial scales, such as quiet backstreets or small pockets of green space and consider relating experienced tranquillity to other measures, for example accessibility.

## Conclusions

In this study we linked experienced tranquillity in landscape to characteristics of landscapes through text data. Based on user-generated descriptions from Geograph we extracted locations described as tranquil across the UK. The spatial patterns of these locations highlighted that people describe tranquillity more often in urban and suburban land cover or broadleaved woodland than expected, but less than expected in e.g. coniferous woodland, heather or bog. Models for the different land cover classes showed different predictors were relevant in different land covers, and an analysis of the texts describing tranquillity in these different land cover types suggests different features contribute to experienced tranquillity depending on the land cover. This study is an example of a hybrid analysis that illustrates the potential of linking individual human experiences described in text with more traditional spatial datasets representing a range of landscape characteristics, through both spatial analysis and a combination of qualitative and quantitative automated text analysis. Our approach provides a way forward in assessing the contributions of landscapes to people across large areas. Text sourced from user-generated content in the form of landscape descriptions brings to the fore public views that are hard to represent in more expert-driven assessment. The methodology we applied showcases a meaningful way for assessing and integrating perspectives and experiences, which is important for harnessing the potential of more inclusive and bottom-up driven landscape planning and policy-making (Edwards 2019). This approach is of relevance to national as well as global assessments such as the Intergovernmental Science-Policy Platform on Biodiversity and Ecosystem Services (IBPES) and nature’s contributions to people (Díaz et al. 2018), since it captures to date hard to represent differences in preference and perception at scale in a broad range of landscapes.

## Electronic supplementary material

Below is the link to the electronic supplementary material.

Supplementary material 1 (PDF 528 kb)

## Footnotes

1 https://www.geograph.org.uk/.

2 e.g.: ‘A perfect place to get some peace and tranquillity’, by margaret ellis (https://www.geograph.org.uk/photo/975294).

3 https://www.rdocumentation.org/packages/stats/versions/3.6.2/topics/glm.

4 https://spacy.io/usage/linguistic-features.

5 https://www.geograph.org.uk/photo/1806677.

6 https://www.geograph.org.uk/photo/3865399.

7 https://www.geograph.org.uk/photo/1037177.

8 https://www.geograph.org.uk/photo/5275128.

9 https://www.geograph.org.uk/photo/507209.

10 https://www.geograph.org.uk/photo/5142640.

## References

[CR1] Antoniou V, Morley J, Haklay M (2010). Web 2.0 geotagged photos: assessing the spatial dimension of the phenomenon. Geomatica.

[CR2] Bieling C (2014). Cultural ecosystem services as revealed through short stories from residents of the SwabianAlb (Germany). Ecosyst Serv.

[CR3] Capineri C (2016). Kilburn high road revisited. Urban Plan.

[CR4] Carver S, Evans AJ, Fritz S (2002). Wilderness attribute mapping in the United Kingdom. Int J Wilderness.

[CR5] Carver S, Watson A, Waters T, Matt R, Gunderson K, Davis B (2009) Developing computer-based participatory approaches to mapping landscape values for landscape and resource management. In: Planning support systems best practice and new methods (pp. 431–448). Springer

[CR6] CEH (2017) Land cover map 2015. Dataset documentation

[CR7] Chesnokova O, Purves RS (2018). From image descriptions to perceived sounds and sources in landscape: analyzing aural experience through text. ApplGeogr.

[CR8] Chesnokova O, Taylor JE, Gregory IN, Purves RS (2019). Hearing the silence: finding the middle ground in the spatial humanities? Extracting and comparing perceived silence and tranquillity in the English Lake District. Int J GeogrInf Sci.

[CR9] Corbane C, Florczyk A, Pesaresi M, Politis P, Syrris V (2018) GHS built-up grid, derived from Landsat, multitemporal (1975-1990-2000-2014), R2018A. European Commission, Joint Research Centre (JRC). 10.2905/jrc-ghsl-10007

[CR10] CPRE (2007) Tranquillity Map: England. Campaign to Protect Rural England

[CR11] Díaz S, Pascual U, Stenseke M, Martín-López B, Watson RT, Molnár Z, Hill R, Chan KMA, Baste IA, Brauman KA, Polasky S, Church A, Lonsdale M, Larigauderie A, Leadley PW, van Oudenhoven APE, van der Plaat F, Schröter M, Lavorel S (2018). Assessing nature’s contributions to people. Science.

[CR12] Donaldson C, Gregory IN, Taylor JE (2017). Locating the beautiful, picturesque, sublime and majestic: spatially analysing the application of aesthetic terminology in descriptions of the English Lake District. J Hist Geogr.

[CR13] Edwards J (2019). Literature and sense of place in UK landscape strategy. Landsc Res.

[CR14] EEA (2016) Quiet areas in Europe—the environment unaffected by noise pollution. EEA Report No 14/2016

[CR15] ESRI (2012) Topographic” [basemap]. Scale Not Given. “World Topographic Map”. February 19, 2012. https://elevation.arcgis.com/arcgis/rest/services/WorldElevation/Terrain/ImageServer

[CR16] Fritz, S., Carver, S., & See, L. (2000). New GIS approaches to wild land mapping in Europe. *In: McCool, SF, Cole, DN, Borrie, WT, O’Loughlin, J. Comps. 2000. Wilderness Science in a Time of Change Conference—Volume 2: Wilderness within the Context of Larger Systems; 1999 May 23–27; Missoula, MT. Proceedings RMRS-P-15*, *15*.

[CR17] Gliozzo G, Pettorelli N, Haklay MM (2016) Using crowdsourced imagery to detect cultural ecosystem services: a case study in South Wales, UK. Ecol Soc 21(3):art6

[CR18] Heikinheimo V, Tenkanen H, Bergroth C, Järv O, Hiippala T, Toivonen T (2020). Understanding the use of urban green spaces from user-generated geographic information. Landsc Urban Plan.

[CR19] Herzog TR, Barnes GJ (1999). Tranquility and preference revisited. J Environ Psychol.

[CR20] Herzog TR, Bosley PJ (1992). Tranquility and preference as affective qualities of natural environments. J Environ Psychol.

[CR21] Hewlett D, Harding L, Munro T, Terradillos A, Wilkinson K (2017). Broadly engaging with tranquillity in protected landscapes: a matter of perspective identified in GIS. Landsc Urban Plan.

[CR22] Hollenstein L, Purves R (2010). Exploring place through user-generated content: Using Flickr to describe city cores. J Spatial Inf Sci.

[CR23] Hosmer DW, Lemeshow S, Klar J (1988). Goodness-of-fit testing for the logistic regression model when the estimated probabilities are small. Biom J.

[CR24] Jackson DL (2000) Guidance on the interpretation of the Biodiversity Broad Habitat Classification (terrestrial and freshwater types): Definitions and the relationship with other habitat classifications. Joint Nature Conservation Committee

[CR25] Jackson S, Fuller D, Dunsford H, Mowbray R, Hext S, MacFarlane R, Haggett C (2008) Tranquillity Mapping: developing a robust methodology for planning support. Report to the Campaign to Protect Rural England, Centre for Environmental & Spatial Analysis, Northumbria University, Bluespace environments and the University of Newcastle upon on Tyne

[CR26] Jeawak SS, Jones CB, Schockaert S (2020). Predicting the environment from social media: a collective classification approach. Comput Environ Urban Syst.

[CR27] Kaplan R, Kaplan S (1989). The experience of nature: a psychological perspective.

[CR28] Koblet O, Purves RS (2020). From online texts to Landscape Character Assessment: Collecting and analysing first-person landscape perception computationally. Landsc Urban Plan.

[CR29] Lake S, Liley D, Still R, Swash A (2015). *Britain’s *habitats: a guide to the wildlife habitats of Britain and Ireland (1st edition).

[CR30] Landis JR, Koch GG (1977). The measurement of observer agreement for categorical data. Biometrics.

[CR31] MacFarlane R, Haggett C, Fuller D, Dunsford H, Carlisle B (2004) Tranquillity Mapping: developing a robust methodology for planning support. Report to the Campaign to Protect Rural England, Countryside Agency, North East Assembly, Northumberland Strategic Partnership, Northumberland National Park Authority and Durham County Council, Centre for Environmental & Spatial Analysis, Northumbria University

[CR32] Manning CD, Schütze H (1999). Foundations of statistical natural language processing.

[CR33] Ministry of Housing Communities and Local Government (2018) National planning policy framework

[CR34] Nugent C, Blanes N, Sáinz de la Maza, M (2016) Quiet areas in Europe: The environment unaffected by noise pollution. In: EEA Report No 14/2016. 10.2800/7586

[CR35] Ortiz-Burgos S, Kennish MJ (2016). Shannon-weaver diversity index. Encyclopedia of earth sciences series.

[CR36] Rowland C, Morton D, Carrasco Tornero L, McShane G, O’Neil A, Wood C (2017a) Land Cover Map 2015 (1km dominant aggregate class, GB)

[CR37] Rowland C, Morton D, Carrasco Tornero L, McShane G, O’Neil A, Wood C (2017b) Land Cover Map 2015 (1km percentage aggregate class, GB).

[CR38] Sappington JM, Longshore KM, Thompson DB (2007). Quantifying landscape ruggedness for animal habitat analysis: a case study using bighorn sheep in the Mojave Desert. J WildlManag.

[CR39] Schiavina M, Freire S, MacManus K (2019) GHS population grid multitemporal (1975, 1990, 2000, 2015) R2019A. Eur Comm JRC 10.2905/42E8BE89-54FF-464E-BE7B-BF9E64DA5218

[CR40] Seresinhe CI, Preis T, MacKerron G, Moat HS (2019). Happiness is greater in more scenic locations. Sci Rep.

[CR41] Seresinhe CI, Preis T, Moat HS (2015) Quantifying the impact of scenic environments on health. Sci Rep 5*(*Article number 16899) 10.1038/srep16899 PMC4658473 26603464

[CR42] Seresinhe CI, Preis T, Moat HS (2017). Using deep learning to quantify the beauty of outdoor places. R Soc Open Sci.

[CR43] Sloan L, Morgan J (2015). Who tweets with their location? Understanding the relationship between demographic characteristics and the use of geoservices and geotagging on Twitter. PLoS ONE.

[CR44] South Downs National Park Authority (2017) Tranquillity study. https://www.southdowns.gov.uk/wp-content/uploads/2017/03/13-04-17-South-Downs-National-Park-Tranquillity-Study.pdf

[CR45] Tenerelli P, Demšar U, Luque S (2016). Crowdsourcing indicators for cultural ecosystem services: a geographically weighted approach for mountain landscapes. Ecol Ind.

[CR46] Tenkanen H, Di Minin E, Heikinheimo V, Hausmann A, Herbst M, Kajala L, Toivonen T (2017). Instagram, Flickr, or Twitter: Assessing the usability of social media data for visitor monitoring in protected areas. Sci Rep.

[CR47] Turner MG (2005). Landscape ecology: what is the state of the science?. Annu Rev EcolEvolSyst.

[CR48] Ulrich RS, Simons RF, Losito BD, Fiorito E, Miles MA, Zelson M (1991). Stress recovery during exposure to natural and urban environments. J Environ Psychol.

[CR49] van Zanten BT, Van Berkel DB, Meentemeyer RK, Smith JW, Tieskens KF, Verburg PH (2016). Continental-scale quantification of landscape values using social media data. Proc Natl Acad Sci USA.

[CR50] Wartmann FM, Mackaness WA (2020). Describing and mapping where people experience tranquillity. An exploration based on interviews and Flickr photographs. Landsc Res.

[CR51] Wartmann FM, Tieskens KF, van Zanten BT, Verburg PH (2019). Exploring tranquillity experienced in landscapes based on social media. ApplGeogr.

[CR52] Watts G, Miah A, Pheasant R (2013). Tranquillity and soundscapes in urban green spaces- predicted and actual assessments from a questionnaire survey. Environ Plan B.

[CR53] Watts GR (2017). The effects of “greening” urban areas on the perceptions of tranquillity. Urban For Urban Green.

[CR54] Watts GR, Pheasant RJ (2015). Tranquillity in the Scottish Highlands and Dartmoor National Park—the importance of soundscapes and emotional factors. ApplAcoust.

[CR55] Watts GR, Pheasant RJ, Horoshenkov KV (2011). Predicting perceived tranquillity in urban parks and open spaces. Environ Plan B.

