# Supplementary material for: Assessing experienced tranquillity through natural language processing and landscape ecology measures
Source: Landsc Ecol. 2021 Jan 27;36(8):2347–65. doi: 10.1007/s10980-020-01181-8 (PMC8550761; doi:10.1007/s10980-020-01181-8)

Supplementary material

Wordclouds for terms grammatically associated with tranquil keywords for different land cover classes

Broadleaved woodland

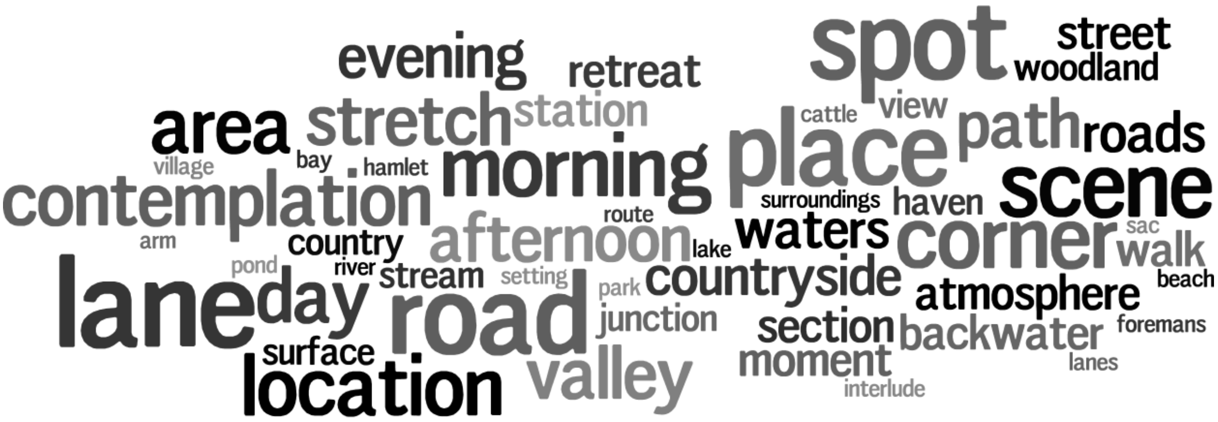

Coniferous woodland

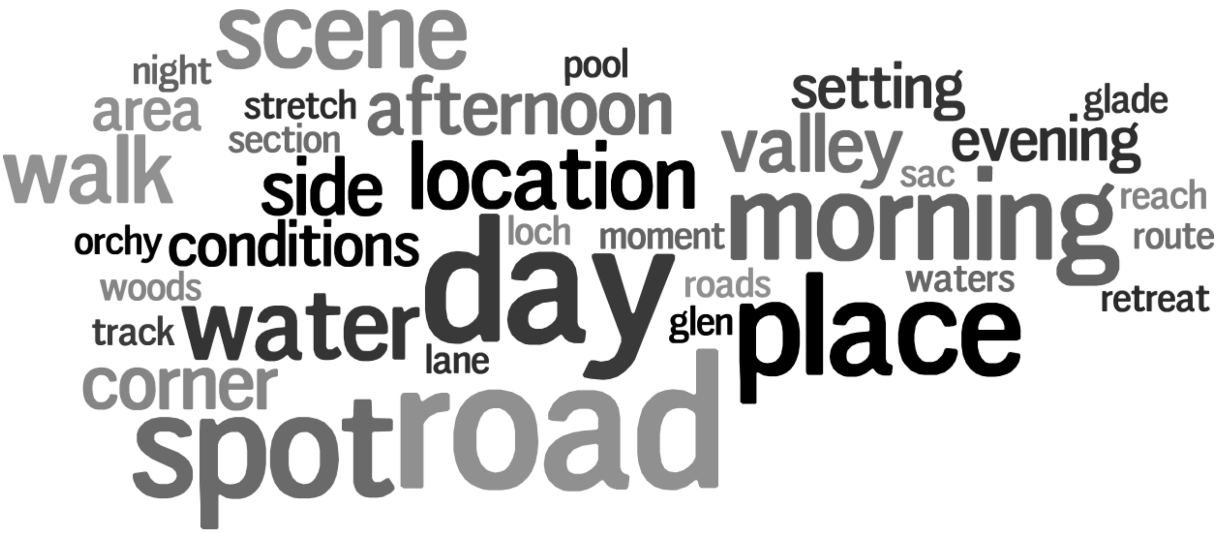

Arable and horticulture

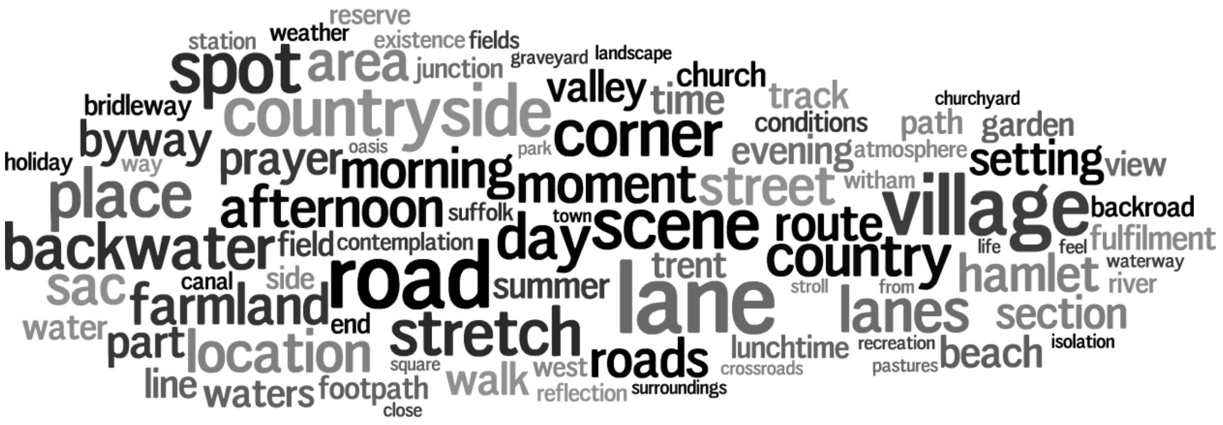

Improved grassland

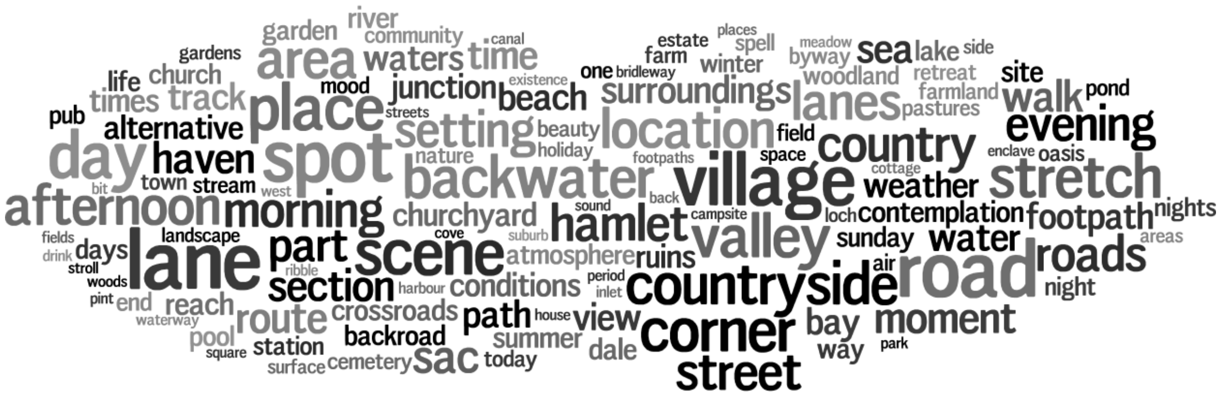

Suburban

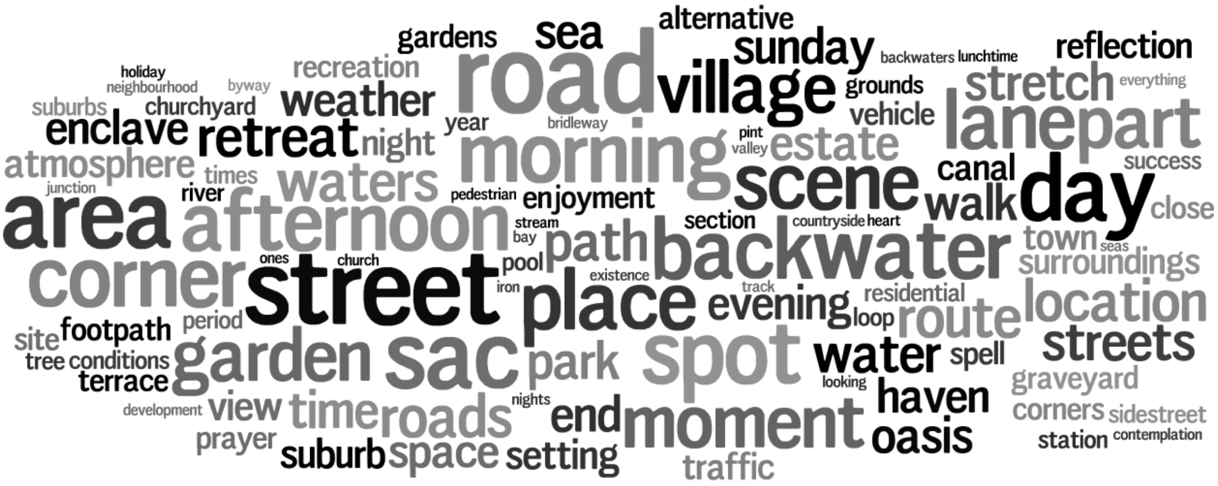

Urban

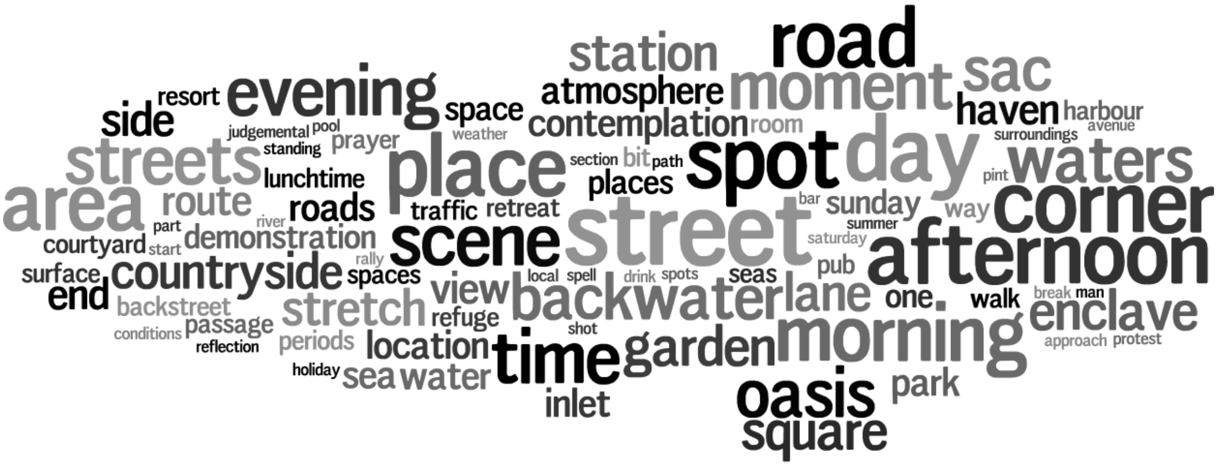

Supplement: Supplementary file 1 — Supplementary material 1 (PDF 528 kb) [file 10980_2020_1181_MOESM1_ESM.pdf]
